# Distinct immune signatures in chronic lymphocytic leukemia and Richter syndrome

**DOI:** 10.1038/s41408-021-00477-5

**Published:** 2021-05-10

**Authors:** Yucai Wang, Sutapa Sinha, Linda E. Wellik, Charla R. Secreto, Karen L. Rech, Timothy G. Call, Sameer A. Parikh, Saad S. Kenderian, Eli Muchtar, Suzanne R. Hayman, Amber B. Koehler, Daniel L. Van Dyke, Jose F. Leis, Susan L. Slager, Haidong Dong, Neil E. Kay, Rong He, Wei Ding

**Affiliations:** 1 Division of Hematology, Mayo Clinic, Rochester, MN USA; 2 Division of Hematopathology, Mayo Clinic, Rochester, MN USA; 3 Division of Laboratory Genetics and Genomics, Mayo Clinic, Rochester, MN USA; 4 Division of Hematology and Medical Oncology, Mayo Clinic, Phoenix, AZ USA; 5 Division of Biomedical Statistics and Informatics, Mayo Clinic, Rochester, MN USA; 6 Department of Immunology, Mayo Clinic, Rochester, MN USA

**Keywords:** Tumour immunology, Chronic lymphocytic leukaemia

## Abstract

Richter syndrome (RS) refers to transformation of chronic lymphocytic leukemia (CLL) to an aggressive lymphoma, most commonly diffuse large B-cell lymphoma. RS is known to be associated with a number of genetic alterations such as *TP53* and *NOTCH1* mutations. However, it is unclear what immune microenvironment changes are associated with RS. In this study, we analyzed expression of immune checkpoint molecules and infiltration of immune cells in nodal samples, and peripheral blood T-cell diversity in 33 CLL and 37 RS patients. Compared to CLL, RS nodal tissue had higher PD-L1 expression in histiocytes and dendritic cells (median 16.6% vs. 2.8%, *P* < 0.01) and PD1 expression in neoplastic B cells (median 26.0% vs. 6.2%, *P* < 0.01), and higher infiltration of FOXP3-positive T cells (median 1.7% vs. 0.4%, *P* < 0.01) and CD163-positive macrophages (median 23.4% vs. 9.1%, *P* < 0.01). In addition, peripheral blood T-cell receptor clonality was significantly lower in RS vs. CLL patients (median [25th–75th], 0.107 [0.070–0.209] vs. 0.233 [0.111–0.406], *P* = 0.046), suggesting that T cells in RS patients were significantly more diverse than in CLL patients. Collectively these data suggest that CLL and RS have distinct immune signatures. Better understanding of the immune microenvironment is essential to improve immunotherapy efficacy in CLL and RS.

## Introduction

Chronic lymphocytic leukemia (CLL) is the most common form of adult leukemia in the Western world^1^. The clinical presentation of CLL is heterogeneous, with a relatively indolent course in a subset of patients. In recent years, the clinical outcome of CLL has improved significantly with the introduction of novel targeted agents such as the Bruton’s tyrosine kinase inhibitor ibrutinib^2–6^ and the B-cell lymphoma 2 inhibitor venetoclax^7–9^. Richter syndrome (RS) refers to the transformation of CLL to an aggressive lymphoma, most commonly diffuse large B-cell lymphoma. RS typically presents aggressively with prominent constitutional symptoms, significant lymphadenopathy, and rapid progression^10–12^. Patients with RS have a poor outcome given the lack of effective therapies, particularly in the era of targeted therapies for CLL. CLL patients who develop RS while on ibrutinib or venetoclax have a median overall survival of ~4–12 months^13–15^.

A number of recurrent genetic abnormalities have been identified in RS, including *TP53* and *NOTCH1* mutations, *CDKN2A* loss, and *MYC* alternations^16^. However, it is unknown whether RS is also associated with changes in the immune microenvironment. In a phase 2 clinical trial (NCT02332980) done at our institution, 4 of the first 9 patients with RS responded to immunotherapy with the PD1-blocking immune checkpoint inhibitor pembrolizumab, but in contrast none of the 16 CLL patients responded^17^. In another phase 2 clinical trial, the combination of another checkpoint inhibitor nivolumab with ibrutinib was also shown to be efficacious in treating RS^18^, with an objective response rate of 42%. These data suggest that the immune microenvironment in patients with CLL and RS may be different.

In this study, we therefore sought to define the immune signatures in CLL and RS, including expression of immune checkpoint molecules (PD-L1, PD1), infiltration of immune cells (T cells, macrophages) in nodal tissue, and T-cell clonality in peripheral blood and lymph nodes. The potential effects of ibrutinib on the immune microenvironment in CLL and RS were also explored.

## Methods

### Patients and samples

This study was approved by Mayo Clinic Institutional Review Board. A total of 33 CLL and 37 RS patients were included in this study. Fifteen CLL patients and 15 RS patients were participants of the NCT02332980 trial. Peripheral blood and nodal biopsy tissue at trial baseline (immediately prior to therapy) were obtained accordingly to the trial protocol and with informed consent. The additional 18 CLL and 22 RS patients were identified from the Mayo Clinic CLL Database^19^, with freshly collected or archived peripheral blood and/or nodal tissue samples available in the Mayo Clinic CLL tissue bank for this study.

### Immunohistochemistry (IHC) staining

Expression of the immune checkpoint molecules in CLL and RS lymph node samples was analyzed by IHC staining using antibodies specific for PD-L1 and PD1. Immune cell infiltration in CLL and RS lymph node samples was assessed by IHC staining using antibodies specific for CD3, CD8, FOXP3, and CD163. IHC staining was done on 4-μm FFPE sections on DAKO Autostainer Plus (Agilent, Santa Clara, CA) using standard protocol^17,20^. The antibodies used include anti-PD-L1 (clone SP263, Ventana Medical Systems, Inc., Tucson, AZ), anti-PD1 (clone NAT105, Abcam, Inc., Cambridge, MA), anti-CD3 (clone LN10, Leica Biosystems, Newcastle Upon Tyne, UK), anti-CD8 (clone C8/144B, Dako, Carpenteria, CA), anti-FOXP3 (clone 236A/E7, Abcam, Inc., Cambridge, MA), and anti-CD163 (clone10D6, Leica Biosystems, Newcastle Upon Tyne, UK). IHC images were taken using whole slide imaging technology with MoticEasyScan Pro (Motic digital pathology, San Francisco, CA), and saved in tagged image file format. Percentage of expression for each individual antigen was calculated by dividing number of cells with positive staining by number of total cells in the image using the Image-Pro premier 3D 9.1.4 software (Media Cybernetics, Silver Spring, MD).

### T-cell clonality

DNA was extracted from T cells that were isolated from peripheral blood mononuclear cells using the EasySep Human CD3 Positive Selection Kit (STEMCELL Technologies, Cambridge, MA**)**, and from lymph node tissue samples as well in a small subset of patients. TRBV gene sequencing was performed using the immunoSEQ assay (hsTCRB kit, Adaptive Biotechnology, Seattle, WA). This assay specifically targets the complementarity determining region 3 (CDR3) of human TRBV gene sequences. CDR3 sequences representing productive rearrangements (in frame) were analyzed while those representing nonproductive rearrangements (out of frame or with a stop codon) were filtered out. T cells with the same productive TCR Vβ CDR3 sequence are considered to comprise a unique T-cell clone. Based on the CDR3 sequences, the TCR Vβ gene usage can be determined for each T-cell clone. In each sample, the fraction of each T-cell clone can be determined by dividing the number of such clonal T cells by the total number of T cells. T-cell clonality depends on both the evenness and richness of a given T-cell population, and is calculated as follows, where *i* represents each individual T-cell clone, Pi represents the fraction of each clone, and *n* represents total number of different clones^21–23^.

$${\mathrm{Clonality}} = 1 - \frac{{ - \mathop {\sum}\nolimits_{i = 1}^n {Pi\left( {{\mathrm{log}}_2Pi} \right)} }}{{{\mathrm{log}}{}_2n}}$$

The calculated T-cell clonality, i.e., TCR clonality, could range from 0 to 1, where a lower clonality indicates a more diverse T-cell population.

### Statistical analysis

The expression of PD-L1, PD1, CD3, CD8, FOXP3, and CD163 was quantified as the percentage of cells with positive staining on IHC images, as descried above. Differences of expression assessed by IHC and T-cell clonality between groups were analyzed using the Wilcoxon rank-sum test and/or unpaired *t*-test. Statistical analysis was done in IBM SPSS Statistics V25. A two-sided *P* value < 0.05 was considered to be statistically significant.

## Results

### Patient cohort

A total of 33 CLL and 37 RS patients were included, and the baseline characteristics (at or near the time of blood or tissue sample collection, on which IHC and T-cell clonality assays were done) were summarized in Table 1. The median age was 69 years (range 44–84) and 67 years (range 42–84), respectively, for the CLL and RS cohorts. Thirteen (39%) CLL and 8 (22%) RS patients were female. Twenty (77%) CLL and 31 (89%) RS patients had unmutated *IgHV*, and 9 (32%) CLL and 14 (45%) RS patients had *TP53* disruption (del(17p) or somatic *TP53* mutation). In the CLL cohort, 20 (61%) patients were treated with chemoimmunotherapy (CIT) only prior to the study, and 13 (39%) patients received CIT and ibrutinib. In the RS cohort, 20 (54%) patients were treated with CIT only, and 17 (46%) patients received ibrutinib treatment (with or without CIT, for RS).

**Table 1:** Baseline characteristics of patients with CLL and RS.

	CLL	RS
Number	33	37
Age, median (range)	69 (44–84)	67 (42–84)
Sex, female	13 (39%)	8 (22%)
Rai stage^a^		
0	3 (10%)	7 (21%)
I–II	10 (32%)	12 (35%)
III–IV	18 (58%)	15 (44%)
*IgHV*, unmutated	20/26 (77%)	31/35 (89%)
CLL FISH		
17p-	8 (29%)	9 (28%)
11q-	4 (14%)	4 (13%)
Trisomy 12	4 (14%)	2 (6%)
Normal	5 (18%)	12 (38%)
13q-	5 (18%)	4 (13%)
Other abnormalities	2 (7%)	1 (3%)
*TP53* disruption	9/28 (32%)	14/31 (45%)
CLL-IPI^a^		
0–1 (low risk)	1 (4%)	0 (0%)
2–3 (intermediate risk)	2 (8%)	6 (19%)
4–6 (high risk)	13 (54%)	11 (35%)
7–10 (very high risk)	8 (33%)	14 (45%)
Prior treatment		
CIT only	20 (61%)	20 (54%)
Ibrutinib^b^	13 (39%)	17 (46%)

*CLL* chronic lymphocytic leukemia, *RS* Richter syndrome, *IGHV* immunoglobulin heavy chain variable region, *FISH* fluorescence in situ hybridization, *IPI* international prognostic index*, CIT* chemoimmunotherapy.

^a^Rai stage and CLL-IPI evaluation were based on evaluation of the CLL component at or near the time of sampling (closest data within 3 years).

^b^Also received CIT for CLL or RS.

### Expression of PD-L1 and PD1

**Fig. 1:**
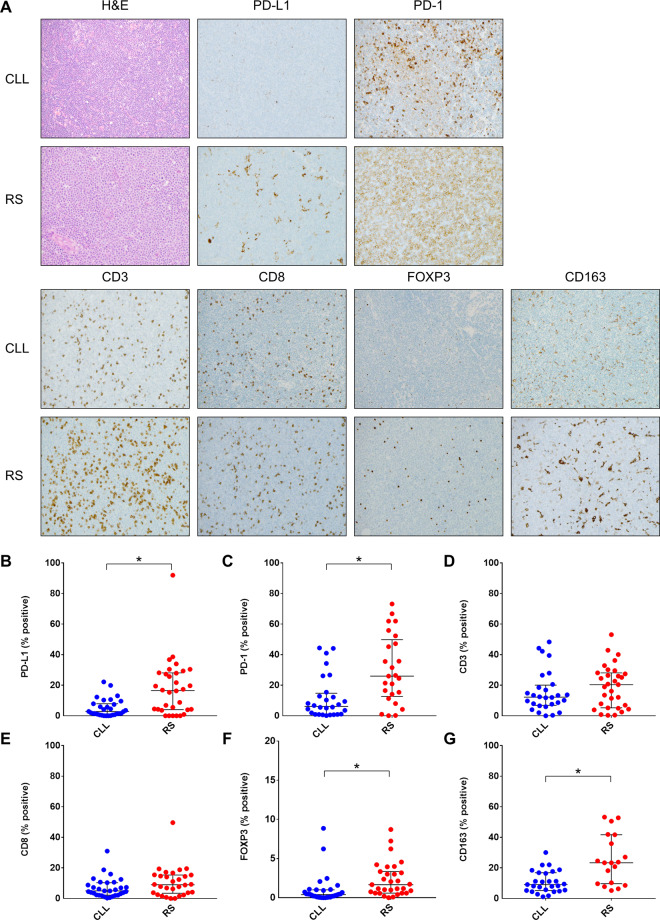
Immunohistochemistry staining of immune checkpoint and immune cell markers in CLL and RS. **A** Representative H&E and IHC images of PD-L1, PD1, CD3, CD8, FOXP3, and CD163 staining in CLL and RS. **B**–**G** Quantitative comparison of PD-L1, PD1, CD3, CD8, FOXP3, and CD163 staining in CLL vs. RS. Asterisk (*) indicates *P* < 0.01.

Representative IHC staining images are shown in Fig. 1A. PD-L1 staining was detected primarily in histiocytes and dendritic cells, while PD1 staining was primarily in neoplastic B cells, consistent with our prior findings^17,24^. As shown in Fig. 1B, the percentage of PD-L1-positive cells was significantly higher in RS (*n* = 32) compared to CLL (*n* = 31) lymph node samples (median 16.6% vs. 2.8%, *P* < 0.01). The percentage of PD1-positive cells was also significantly higher in RS (*n* = 25) compared to CLL (*n* = 28; Fig. 1C) lymph node samples (median 26.0% vs. 6.2%, *P* < 0.01). In both the CLL and RS cohorts, the expression of PD-L1 and PD1 was similar in lymph node samples from patients exposed to CIT only vs. those exposed to ibrutinib (Table 2).

**Table 2:** Quantified results of immunohistochemistry staining in CLL and RS samples.

	CLL		RS		
	*N*	Median (25th, 75th)	*N*	Median (25th, 75th)	*P*
PD-L1	31	2.8 (0.8, 7.9)	32	16.6 (4.0, 28.1)	<0.01
PD1	28	6.2 (1.0, 14.8)	25	26.0 (12.7, 49.8)	<0.01
CD3	28	12.2 (6.8, 20.1)	31	20.4 (5.2, 28.1)	0.32
CD8	31	5.0 (2.2, 10.3)	30	9.0 (3.4, 15.4)	0.09
FOXP3	32	0.4 (0.1, 1.0)	31	1.7 (0.6, 3.3)	<0.01
CD163	27	9.1 (5.2, 16.9)	19	23.4 (9.6, 41.7)	<0.01
	**CLL post-CIT**		**CLL post-ibrutinib**		
PD-L1	18	1.6 (0.4, 6.9)	13	4.5 (1.5, 8.8)	0.21
PD1	16	5.4 (0.9, 10.1)	12	10.2 (1.9, 39.3)	0.16
CD3	16	10.8 (6.1, 14.4)	12	15.3 (8.2, 41.6)	0.12
CD8	18	4.7 (2.2, 9.2)	13	5.0 (2.3, 11.6)	0.77
FOXP3	19	0.3 (0.1, 0.7)	13	0.4 (0.2, 1.6)	0.18
CD163	16	7.8 (4.5, 11.2)	11	11.2 (7.9, 18.8)	0.09
	**RS post-CIT**		**RS post-ibrutinib**		
PD-L1	15	16.8 (4.3, 28.2)	17	15.4 (1.8, 28.6)	0.68
PD1	11	15.4 (8.0, 26.5)	14	35.6 (20.2, 57.4)	0.07
CD3	14	22.6 (7.1, 28.5)	17	16.2 (4.5, 29.6)	0.68
CD8	14	9.0 (5.6, 14.2)	16	8.5 (2.3, 16.8)	0.93
FOXP3	16	1.7 (0.7, 3.8)	15	1.2 (0.6, 3.3)	0.89
CD163	11	23.8 (10.3, 41.9)	8	20.7 (7.1, 33.7)	0.35

Percentage of expression for each individual antigen was calculated by dividing number of cells with positive staining by number of total cells in the image.

*CLL* chronic lymphocytic leukemia, *RS* Richter syndrome, *CIT* chemoimmunotherapy.

### Infiltration of immune cells

Infiltration levels, as assessed by IHC, of CD3 or CD8-positive T cells were similar in CLL and RS lymph node samples (Fig. 1D, E). However, RS samples (*n* = 31) had more infiltration of FOXP3-positive T cells compared to CLL samples (*n* = 32; median 1.7% vs. 0.4%, *P* < 0.01; Fig. 1F). In addition, RS samples (*n* = 19) also had higher infiltration of CD163-positive macrophages compared to CLL samples (*n* = 27; median 23.4% vs. 9.1%, *P* < 0.01; Fig. 1G). In both the CLL and RS cohorts, the infiltration levels of CD3-positive T cells, CD8-positive T cells, FOXP3-positive T cells, as well as CD163-positive macrophages were similar in lymph node samples from patients exposed to CIT only vs. those exposed to ibrutinib (Table 2).

### T-cell clonality

**Fig. 2:**
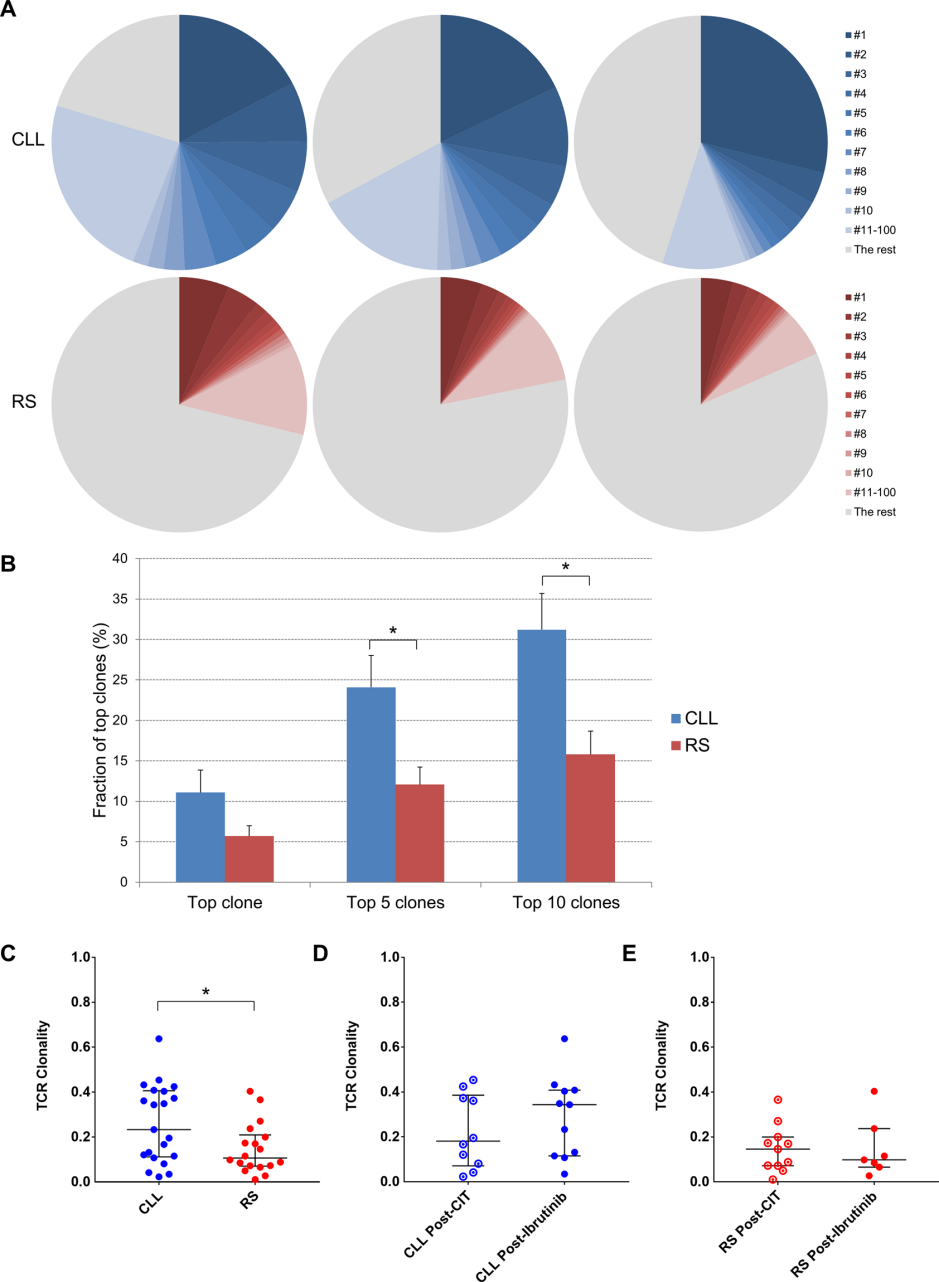
Peripheral blood TCR clonality in CLL and RS patients. **A** Fractions of top 10 and top 100 TCR clones in representative CLL (*n* = 3, upper panel) and RS (*n* = 3, lower panel) cases. **B** Quantitative comparison of top 1, top 5, and top 10 TCR clone fractions in CLL (*n* = 21) and RS (*n* = 18) cases. **C** TCR clonality in CLL vs. RS cases. **D** TCR clonality in post-CIT vs. post-ibrutinib CLL cases. **E** TCR clonality in post-CIT vs. post-ibrutinib RS cases. Asterisk (*) indicates *P* < 0.05.

Representative images showing clonal distribution of peripheral blood T cells are shown in Fig. 2A. Examples of three CLL and three RS cases are displayed, showing the fractions of top 10 as well as top 11–100 clones combined in each sample. There tended to be fewer unique T-cell clones in CLL vs. RS samples, and the leading clones in CLL samples tended to comprise larger fractions among the total T-cell population. As shown in Fig. 2B, the fractions of the top five and ten clones were significantly larger in CLL samples (*n* = 21) compared to RS samples (*n* = 18; 24.1 ± 3.9% vs. 12.1 ± 2.2% for top five clones combined, *P* = 0.015; 31.2 ± 4.5% vs. 15.8 ± 2.9% for top ten clones combined, *P* = 0.008). As shown in Fig. 2C, peripheral blood T-cell TCR clonality was significantly lower in RS (*n* = 18) vs. CLL (*n* = 21) patients (median [25th–75th], 0.107 [0.070–0.209] vs. 0.233 [0.111–0.406], *P* = 0.046). In a subset of RS patients (*n* = 4) with paired peripheral blood and nodal tissue available, TCR clonality scores were identical between paired samples (data not shown). Collectively the T-cell clonality data suggest that T cells in CLL patients were less diverse compared to RS patients.

CLL patients with prior exposure to CIT only (*n* = 10) or exposure to ibrutinib (*n* = 11) had similar TCR clonality (median [25th–75th], 0.181 [0.071–0.386] vs. 0.343 [0.115–0.408], *P* = 0.56; Fig. 2D). RS patients with prior exposure to CIT only (*n* = 11) or exposure to ibrutinib (*n* = 7) also had similar TCR clonality (median [25th–75th], 0.146 [0.072–0.200] vs. 0.098 [0.066–0.237], *P* = 0.93; Fig. 2E). This information indicated that the T-cell diversity status in RS and CLL may be independent of the type of prior therapy.

### TCR Vβ gene usage

**Fig. 3:**
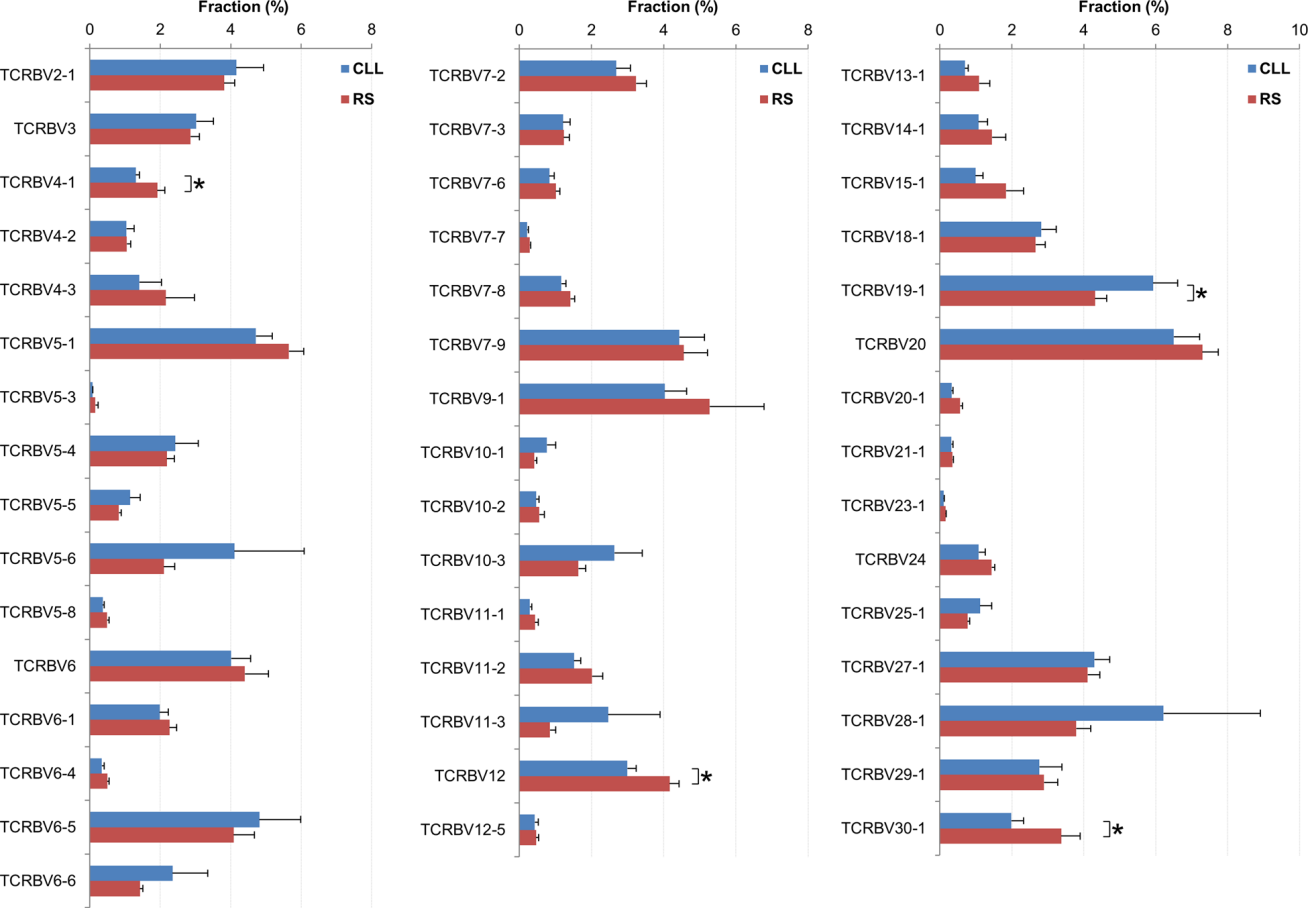
Distribution of TCR Vβ gene usage in CLL (*n* = 21) vs. RS (*n* = 18) cases. Genes with <0.1% fraction in both CLL and RS cases were not illustrated. Asterisk (*) indicates *P* < 0.05.

The distribution of TCR Vβ gene usage among the T cells in CLL (*n* = 21) and RS (*n* = 18) patients are shown in Fig. 3. Overall the distribution patterns appeared similar between CLL and RS patients. The fractions of clones with several particular TCR Vβ genes did show a statistical difference between CLL and RS patients. For example, compared with RS, CLL samples had lower TRBV4-1 (1.31 ± 0.10% vs. 1.92 ± 0.21%, *P* = 0.009), TRBV12 (2.99 ± 0.24% vs. 4.16 ± 0.27%, *P* = 0.002), and TRBV30-1 (1.99 ± 0.34% vs. 3.37 ± 0.53%, *P* = 0.029) usage, but higher TRBV19-1 (5.93 ± 0.68% vs. 4.31 ± 0.32%, *P* = 0.049) usage. However, it is unclear whether there is any clinical significance given the small absolute differences.

## Discussion

In this study, we demonstrated that CLL and RS have distinct immune signatures in both the lymph nodes and peripheral blood. Patients with RS had a lower peripheral blood T-cell TCR clonality and more diverse T-cell repertoire compared to CLL patients. In addition, we found that expression of PD-L1 in histiocytes and PD1 in neoplastic B cells, as well as infiltration of FOXP3-positive T cells and CD163-positive macrophages were increased in RS compared to CLL lymph node samples. These unique findings emphasize the different immune microenvironments in CLL and RS and may partially explain the different responses to therapy with immune checkpoint inhibitors^17^.

In solid tumors such as melanoma and lung cancer and classical Hodgkin lymphoma, increased expression of PD-L1 by the malignant cells and PD1 by T cells lead to T-cell exhaustion and immunosuppression^25^. Blocking the PD-L1/PD1 immune checkpoint signaling with anti-PD-L1/PD1 antibodies to activate tumor reactive cytotoxicity has proven efficacious in treating these malignancies^25^. Teng et al.^26,27^ proposed a model to classify tumor microenvironments based on PD-L1 expression in tumor cells and tumor infiltration lymphocytes (TIL), i.e., Type I, PD-L1+, TIL+, indicating adaptive immune resistance; Type II, PD-L1−, TIL−, indicating immunological ignorance; Type III, PD-L1+, TIL−, indicating intrinsic induction; and Type IV, PD-L1−, TIL+, indicating immune tolerance, likely via other suppressors (e.g., checkpoint pathways other than PD-L1/PD1). Interestingly, in CLL and RS, PD-L1 staining is primarily on histiocytes and dendritic cells, while PD1 staining is primarily on malignant B cells^17,24^. CLL likely has a Type II tumor microenvironment with immunological ignorance and will unlikely respond to immune checkpoint blockade. RS may have a Type I tumor microenvironment with both PD-L1 expression and TILs and immunotherapy is therefore potentially effective. However, one may also argue that RS has a Type IV tumor microenvironment, as the PD-L1 expression is primarily on histiocytes and dendritic cells instead of tumor B cells; therefore, immunotherapy strategies in addition to PD1 blockade may be necessary.

Our prior study showed that there is a progressive increase of neoplastic B-cell expression of PD1 from prolymphocytes and paraimmunoblasts in CLL proliferation centers to the large B cells in RS^24^. Our current study demonstrated that the staining of PD-L1 (in histiocytes and dendritic cells) and PD1 (in neoplastic B cells) was significantly higher in RS compared to CLL samples. The exact mechanism and the functional impact of PD1 expression in the malignant B cells in CLL and RS are unclear at this time. While immune checkpoint inhibitors can block PD-L1/PD1 signaling and reactivate T-cell immunity, we postulate that they might also have direct cytotoxic effects toward neoplastic B cells in CLL and RS, much more pronounced in the latter due to higher expression of PD-L1/PD1.

A number of T-cell abnormalities have previously been reported in CLL, such as increased absolute numbers, inversed CD4:CD8 ratio, and prominent oligoclonality^28–31^. The mechanism and impact of T-cell oligoclonality in CLL are unclear. In this study we demonstrated a lower clonality, thus higher diversity, of peripheral blood T cells in RS patients. This might have implications in aiding in detection of RS as well, as progressively decreasing peripheral blood T-cell clonality may represent a unique biomarker of CLL transformation. However, our findings here need to be further validated, ideally in prospective studies, with serial monitoring of blood and lymph nodes where feasible.

What drives the diversification of T cells in Richter transformation is unclear. One possibility is the formation of neoantigens. RS is associated with multiple genetic alterations including those involving *TP53*, *NOTCH1 CDKN2A*, and *MYC*^16^. These alterations likely result in genomic instability and increased genetic mutations. As a result, there may be increased neoantigen generation in RS. In this setting, the increased infiltration of immune cells and diversity of T cells in RS is likely a reflection of host immune responses. Thus immunotherapy with PD1/PD-L1 inhibitors can block the immune checkpoint and reactivate T cells. The diversity of T cells has been recognized to be important for effective immune response upon reactivation^32^. Solid tumors with mismatch repair deficiency or high tumor mutation burden presumably contain more tumor antigens, and effective immunotherapy in these cases likely relies on a diversity of T cells that recognize different antigens^33^. The lower T-cell clonality and thus higher diversity in RS may partially explain our detection of superior clinical responses to immunotherapy compared to CLL.

Of note, responses to pembrolizumab were seen in RS patients with prior exposure to ibrutinib but not patients treated with CIT only^17^; however, our current study did not show significant differences in PD-L1/PD1 expression, immune cell infiltration, or T-cell clonality between RS patients exposed to ibrutinib or CIT only. Whether ibrutinib exposure affects the immune microenvironment in CLL and RS remains to be investigated in the future. We believe it should as this drug has the potential of altering both the T-cell status of treated patients as well as altering the biology of the neoplastic B cells and thus also impacting on the response to immunotherapy.

In summary, we demonstrated clear differences in the immune signature of CLL and RS in blood and nodal tissue sites. Better understanding of the progressive changes in immune microenvironment associated with CLL transformation to RS is likely important given this new information. Studies on mutational burden and neoantigen generation associated with the transformation may help fill the knowledge gaps. Clinical trial results do support targeting immune checkpoint in RS treatment^17,18^, but identifying biomarkers such as changes in T-cell diversity that can predict clinical responses will be important.
